# Settling velocities of coarse organic solids

**DOI:** 10.1038/s41598-023-39645-x

**Published:** 2023-08-01

**Authors:** Aaron J. Pietsch, John A. Chapman

**Affiliations:** Department of Bioproducts and Biosystems Engineering, University of Minnesota, Twin Cities, 1390 Eckles Ave, Saint Paul, MN 55108 USA

**Keywords:** Fluid dynamics, Hydrology

## Abstract

The settling velocity of a particle is an integral parameter in stormwater modeling and design. The settling velocity can be used to predict the fate and transport of stormwater particles and if the particles contribute to nutrient loading in a watershed. Prediction of settling velocity for inorganic particles is generally well-researched and well-understood. Organic particles tend to vary widely in their physical properties and there are currently no set standards or empirical equations for estimating the settling velocity of organic particles. This paper presents data from tree leaves and seeds settling velocity experiments to better understand how organic particles settle in the context of settling velocity equations such as the one developed by Ferguson and Church. Analysis of the collected data showed that the second of the two drag coefficients (C_2_) used in the Ferguson and Church Equation was sensitive to particle type and shape. By averaging C_2_ by particle type and species, there was a correlation between the observed settling velocity and the settling velocity predicted by the Ferguson and Church Equation (R^2^ = 0.83). With these results, stormwater modelers and designers are equipped with a better understanding of how to represent common organic particles in terms of settling velocity. Additional research on a wider variety of organic particle types and species would expand on the dataset presented here.

## Introduction

Urbanization has led to unique challenges in water management in cities dominated by impervious surfaces. The need to treat, store, and infiltrate water for the benefit of humans and the environment is paramount as urban populations continue to grow and climate change intensifies weather events^1^. Stormwater, rainwater that runs off impervious surfaces, is known as a particularly broad conveyor of pollutants. Stormwater carries chemical, physical, and biological pollutants into receiving water, degrading water quality and aquatic habitat^2^.

Stormwater is treated using Stormwater Control Measures (SCMs), which are structures designed to infiltrate, store, or treat stormwater runoff based on basic scientific principles^3^. In urban residential areas, coarse inorganic and organic debris are pollutants of high concern^2^. Coarse organic and inorganic particles consist of metals^4^, sediment with bound nutrients^5^, and materials that leach nutrients^6^.

Many urban residential SCMs rely on sedimentation to treat stormwater^3^, making settling velocity a key parameter in design and evaluation. For example, stormwater basins are a very common SCM found along roadways and parking lots. Stormwater basins lower total suspended solids and total phosphorus entering downstream water bodies by storing the stormwater and allowing sediment to settle to the bottom of the basin before releasing the water at the outlet^7^.

Settling velocity (w) is a physical property of particles that depends on the particle’s size, shape, and density. Settling velocity for a range of particle sizes may be used in stormwater treatment modeling to estimate the trapping efficiency of basins^8^. Sedimentation may also be estimated with a range of settling velocity values, and this info is needed to inform how soon an infiltration basin would fill up and need to be dredged.

Several studies explore and predict nonspherical mineral particle settling velocities^9–12^. Settling velocity can be calculated for particles with a known diameter (D) and specific gravity (SG) using Stokes’ Law^13^. Stokes’ Law calculations assume that the falling particle is spherical, which is generally valid for mineral or sediment particles. Drag coefficients C_1_ (applicable to laminar flow) and C_2_ (applicable to turbulent flow) can be adjusted to predict nonspherical mineral settling velocities^11^.

Relatively few studies have addressed settling velocities of organic particles, which are also much less likely to be spherical in shape. Shapes of organic particles vary widely from thin, flat tree leaves to prismatic-shaped wood chips and everything in between. The internal structure and how to represent the specific gravity of organic material^14^ further complicate application of mineral settling velocity equations. In this study, the physical properties and settling velocities of nonspherical organic particles will be measured and fit to a predictive equation produced from earlier research on mineral particles.

## Experimental methods

Settling velocity experiments were conducted in the Environmental Engineering Lab in the Biosystems and Agricultural Engineering Building on the St. Paul campus of the University of Minnesota. Inorganic mineral particles and organic particles were selected from samples collected as part of a stormwater solids accumulation research project in 2021. As part of the stormwater solids project, the samples were homogenized, split into smaller representative samples, and stored in a freezer for settling velocity analysis. The most commonly observed tree species in the samples were chosen for settling velocity analysis. The three tree species used in the experiment were Quercus rubra (Red Oak), Acer platanoides (Norway Maple), and Ulmus americana (American Elm). Wood chips were included in the experiments, but not included in the analysis due to their deviation in shape from seeds and leaves. Additionally, some wood chip particles never became saturated enough to sink and could not be included in the settling velocity analysis. The organic particles were taken from a range of collection dates and differed in type, level of decomposition, and physical properties. Particle types are shown in Table 1. The maximum diameter ranges for each particle type are as follows: 9.89 to 18.9 cm for Red Oak leaves, 6.46 to 16.1 cm for Norway Maple leaves, 3.11 to 5.63 cm for Norway Maple seeds, and 1.04 to 1.38 cm for American Elm seeds. Detailed physical properties of the particles are included in the Supplementary Information attachment.

**Table 1:** Particles included in settling velocity experiments.

Particle type	Number of particles
American Elm seed	12
Norway Maple seed	12
Red Oak leaf	12
Norway Maple leaf	12
Wood chips	8

Organic particles were selected from stormwater solids samples collected in Minneapolis and St. Paul, Minnesota, in 2021.

Plant material collected for the settling velocity experiments was collected from cultivated boulevard trees planted by the City of St. Paul, the City of Shoreview, or the University of Minnesota. Permission from each entity was obtained prior to collection. No permits or licenses were required to collect plant material that fell from the cultivated trees into the curb. The authors complied with relevant institutional, national, and international guidelines and legislation regarding plant material collection. The City of St. Paul provided a map of city-planted boulevard trees, which was used to identify the species of trees from which plant material was collected. For sites in Shoreview and on the University of Minnesota campus, Aaron Pietsch provided identification of tree species using a dichotomous key from Chadde^15^. Voucher samples were not sent to an herbarium.

**Figure 1:**
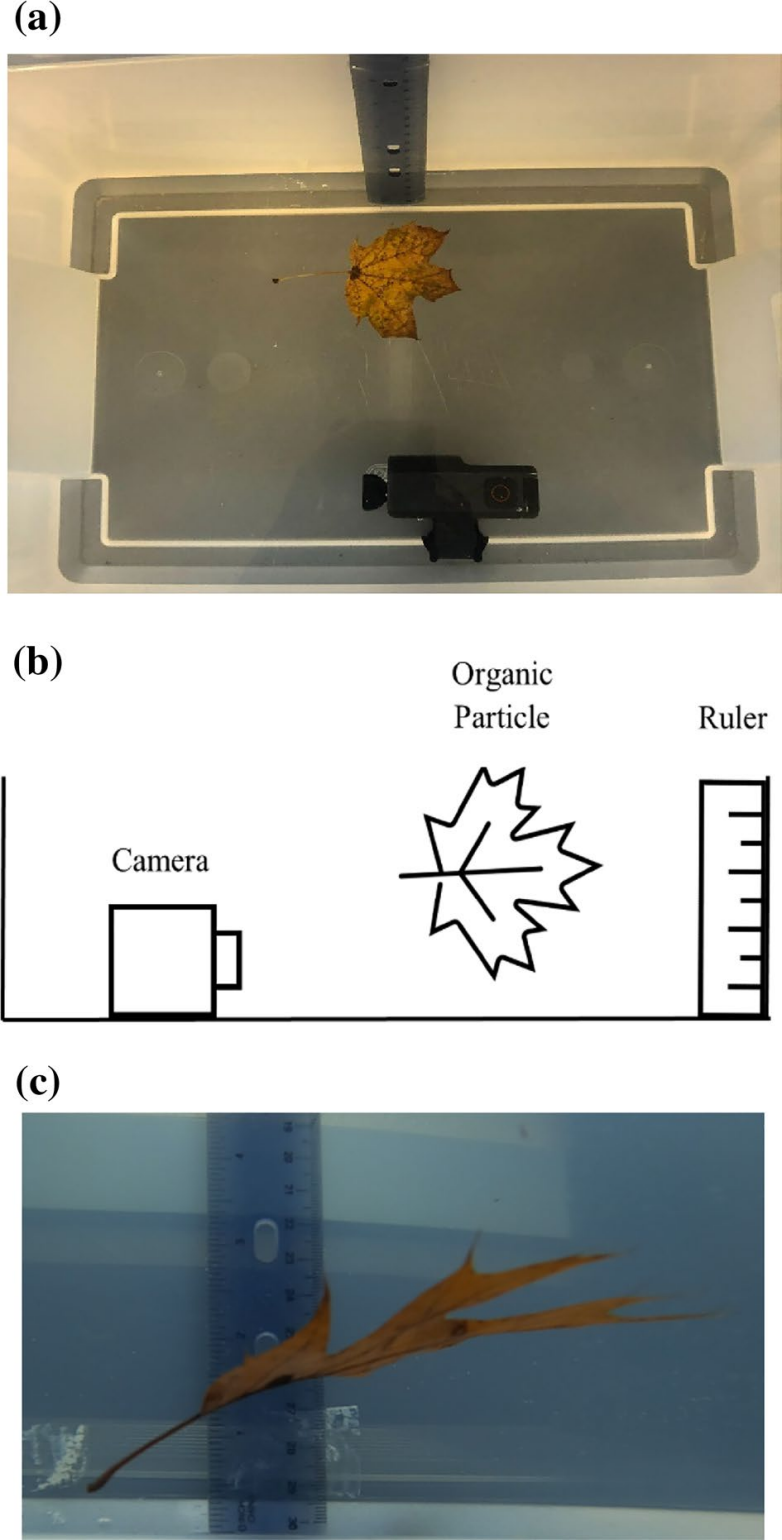
Plan (**a**) and profile (**b**) views of the experimental setup. (**c**) Shows a still from one of the video files used for analysis.

A 19-L tank was filled with tap water and equipped with a ruler to serve as the experimental settling tank (Fig. 1). The water allowed to reach ambient temperature (approximately 22 °C) before experimentation began. Particles were dropped three times each in the tank and filmed by a waterproof GoPro Hero 8 camera at 120 frames/s for fast-falling particles (≥ 0.5 m/s) and 60 frames/s for slow-falling particles (< 0.5 m/s). Organic particles generally did not sink immediately after placement into the settling tank and needed to be pre-saturated before the settling velocity could be measured. Pre-saturation times ranged from 1 to 5 days, depending on the particle type, size, and the physical condition of the particles. Settling velocities were calculated assuming clean particles, or particles that were not affected by external conditions.

The video files were analyzed using Tracker© software to measure point mass position frame-by-frame. The vertical position and time data from the software was used to calculate the settling velocity of each particle. One particle was settled at a time, so the type of settling observed was discrete settling for all particles. To assess the effects of multiple particles settling together, such as compression or hindered settling, further research is required.

**Figure 2:**
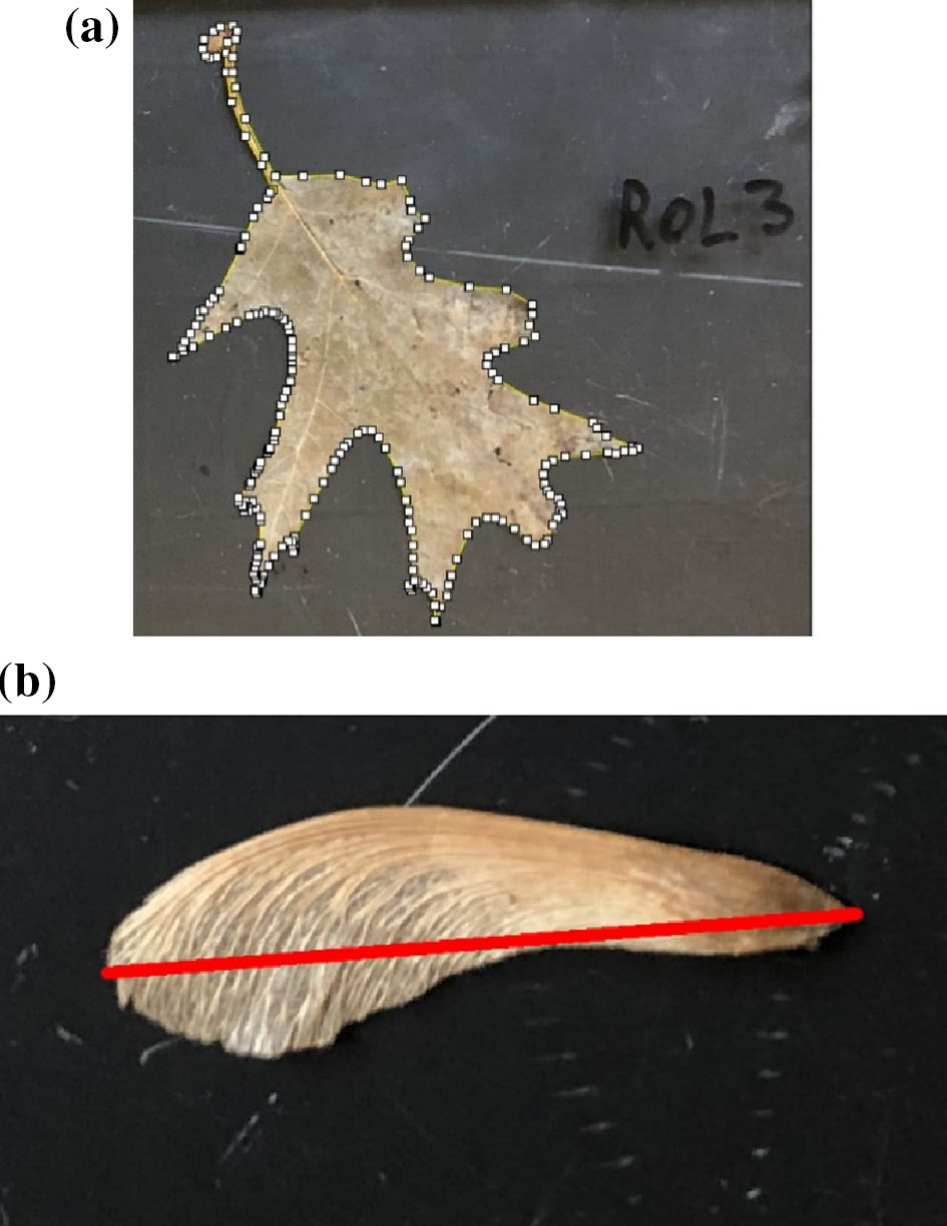
Analysis of (**a**) surface area and (**b**) F_max_ of organic particles using ImageJ software and used to estimate settling velocities in Eqs. (1) and (2).

The maximum Feret diameter (F_max_) is defined by Walton^16^ as the perpendicular distance between parallel tangents touching opposite sides of the profile, which can also be defined as: the longest distance across the profile of a particle as measured with calipers (Fig. 2b). F_max_ has been used to analyze particle shapes and particle size distributions from digital imagery in past studies, especially for irregular shapes^17,18^.

The SG of mineral particles is a straightforward measurement and there are tight ranges reported for minerals, with 2.6 used as a typical value^19^. Mineral particles are solid matter but leaves and other vegetative matter have internal air and liquid components because of their cellular structure^20^. A measure of SG for organic particles, therefore, is a bulk measure of SG that includes water and air in the cellular void spaces. In this study, SG_bulk_ will be used to indicate the bulk specific gravity of organic particles including internal air and liquid components, which was determined by dividing the measured mass by the particle volume displacement in water. From our measurements and by assuming cellulose material has a density of 1.5 g/cm^3^ we were able to estimate the amount of void space to be filled with water that would result in a SG_bulk_ of 1.05, or a point that should induce settling. These values are denoted as SG_1.05_ and resulted in a void saturation of 77% to 102% by volume with an average of 91% and a standard deviation of 8.2. Settling would likely begin once the SG_bulk_ reached a value larger than 1 even though the void spaces were not fully saturated. These assumptions do not account for surface tension effects that could prevent settling of small particles that are denser than water, but the consistency suggests the assumptions are reasonable.

Reynold’s number (Re) describes the relative magnitude of viscous and inertial forces of an object moving through a liquid^10^. Re values were calculated for all particles included in this study and statistics are shown in Table 2. The Re results indicate that American Elm seeds fell in intermediate flow regimes and the rest of the particles fell under turbulent regimes. Recent studies on microplastic settling velocity have observed Re ranging from approximately 100 to 1,000^21^. Only American Elm seeds fell within this range, with the Re of the rest of the seeds and leaves exceeding 1000. This is due to the larger particle diameters used in this study compared to other studies using small-diameter (< 0.005 m) natural grains or microplastics.

**Table 2:** Re statistics for all particles.

Particle type	Average Re	Standard deviation of Re
American Elm seed	202	69
Norway Maple seed	4344	2762
Red Oak leaf	3737	1294
Norway Maple leaf	2117	1006
All seeds	2273	2847
All leaves	2927	1414

An empirical equation to predict the settling velocity of nonspherical soil particles was developed by Kim et al.^22^. The study measured several shape properties of the soil particles and produced the equation based on the best fit of all the variables as determined by nonlinear regression. The equation is based on three diameter measures and is as follows:

1 $$V=14.5\sqrt{{D}_{mean}\frac{{D}_{min}}{{D}_{max}}}$$

where V is the settling velocity (LT^−1^), D_min_ is the minimum Feret diameter (L), D_max_ is the maximum Feret diameter (L), and D_mean_ is the average of D_min_ and D_max_ (L). In this study, Eq. (1) was used as a predictive equation for the settling velocity of the 48 organic particles. The predicted settling velocities from Eq. (1), however, showed no significant correlation with the observed settling velocities.

A shape factor used in natural grain settling velocity studies is aspect ratio^22^. The aspect ratio is simply the ratio of the maximum diameter (referred to as F_max_ in this study) to the minimum diameter in two dimensions. The greater the aspect ratio of a particle, the more elongated the particle is. The aspect ratio for each particle was calculated and statistics are shown in Table 3. The aspect ratio results suggest that most particles were not elongated: American Elm seeds, Red Oak leaves, and Norway Maple leaves were all about 1.5 times as long as they were wide. The aspect ratio of Norway Maple seeds, however, indicate that they were elongated as they were over three times as long as they were wide.

**Table 3:** Aspect ratio statistics for all particles.

Particle type	Average aspect ratio	Standard deviation of aspect ratio
American Elm seed	1.65	0.16
Norway Maple seed	3.22	0.33
Red Oak leaf	1.75	0.25
Norway Maple leaf	1.58	0.25
All seeds	2.43	0.83
All leaves	1.67	0.26

Ferguson and Church^11^ presented an explicit equation derived from Stokes’ Law with two drag coefficients (Eq. 2). This study used Eq. (2) to estimate drag coefficients (C_1_ and C_2_) for organic particles after the settling velocity of the particles had been measured in a settling tank. Equation (2) showed positive correlation (R^2^ = 0.83) between the predicted and observed settling velocities.

2 $$w=\frac{SGg{D}^{2}}{{C}_{1}\nu + {\left(0.75{C}_{2}SGg{D}^{3}\right)}^{0.5}}$$

where w is the settling velocity (LT^−1^), SG is the specific gravity of the particle (dimensionless), D is the diameter of the particle (L), ν is the kinematic viscosity of the fluid (L^2^T^−1^), and g is the gravitational constant (LT^−2^). In this study, SG_bulk_ and SG_1.05_ were both used for SG as indicated and F_max_ is used for D in Eq. (2).

Ferguson and Church^11^ used the Corey Shape Factor (CSF) to represent spherical particle shapes and Goral et al.^21^ used CSF to represent regular and irregular microplastic shapes. The particles used in this study, however, were similar to each other in shape: wide in two dimensions and relatively thin in the third, perpendicular dimension. The CSF was low and did not vary from particle to particle as much as F_max_ (measured CSF for Maple Leaves ranged from 0.00275 to 0.00467). F_max_ was chosen to represent the diameter used in settling velocity equations instead of CSF because F_max_ captures the variability of the size of the particles in two dimensions. Additionally, F_max_ was the most reliably determined measurement from photos of particles. The minimum diameter of the particles was not appropriate to represent the diameter used in settling velocity equations because the minimum diameter measured the width of the petiole of leaves, which is lacking in the seeds. The petioles skewed the minimum diameter (and therefore the average of the minimum and maximum diameters) of leaves.

ImageJ software was used to measure F_max_ and surface area of the particles (Fig. 2). The volume of organic particles was measured by submerging the saturated particle in a graduated cylinder with a known volume of water and measuring the change in volume.

### Plant material collection

Plant material (tree leaves and seeds) was collected for this project within applicable city ordinances, state laws, and federal laws. The collected plant material did not contain any endangered/threatened species or state prohibited noxious weeds.

## Results and discussion

Iteration was used to find C_1_ and C_2_ values in Eq. (2) by calculating the predicted settling velocity based on physical properties of each particle and setting the calculated settling velocity to the observed settling velocity and solving for C_1_ and C_2_. Initial sensitivity analyses suggested that C_2_ was the more influential parameter in the data and C_1_ was set to a value of 100 for all analyses. Goral et al.^21^ found that microplastics of the same shape, flat disks and square plates, had a constant drag coefficient of about 1.23 at Re less than 1000. Ferguson and Church^11^ describe C_1_ as the constant in Stokes’ equation for laminar settling and C_2_ as the constant for Re greater than 1000. The particles tested in this study were all in intermediate or turbulent flow regimes (Re > 1000), so it is logical that C_2_ was more influential to the settling velocity predictive equation.

Averaged C_2_ values using initial unsaturated SG_bulk_ values are shown in Table 4 and averaged C_2_ values using an SG_bulk_ value of 1.05 are shown in Table 5. Although out of the range of the suggested values from Ferguson and Church^11^ (18 to 24 for C_1_ and 0.4 to 1.2 for C_2_), the C_2_ values had a relationship to the F_max_ for seeds, as is discussed later in this section.

**Table 4:** C_2_ values and settling velocities for all particles using dry SG values.

Particle type	Average C_2_ value	Standard deviation of C_2_	Average observed settling velocity (cm/s)
American Elm seed	154	55	1.56
Norway Maple seed	27	23	8.42
Red Oak leaf	1673	732	2.33
Norway Maple leaf	1534	946	1.89
All seeds	91	76	4.99
All leaves	1636	852	2.14

Tree seeds tended to fall faster and have a lower C_2_ value than tree leaves.

**Table 5:** C_2_ values for all particles using an SG value of 1.05.

Particle type	Average C_2_ value	Standard deviation of C_2_
American Elm seed	881	471
Norway Maple seed	178	147
Red Oak leaf	3926	1840
Norway Maple leaf	4942	2597
All seeds	530	495
All leaves	4434	2307

**Figure 3:**
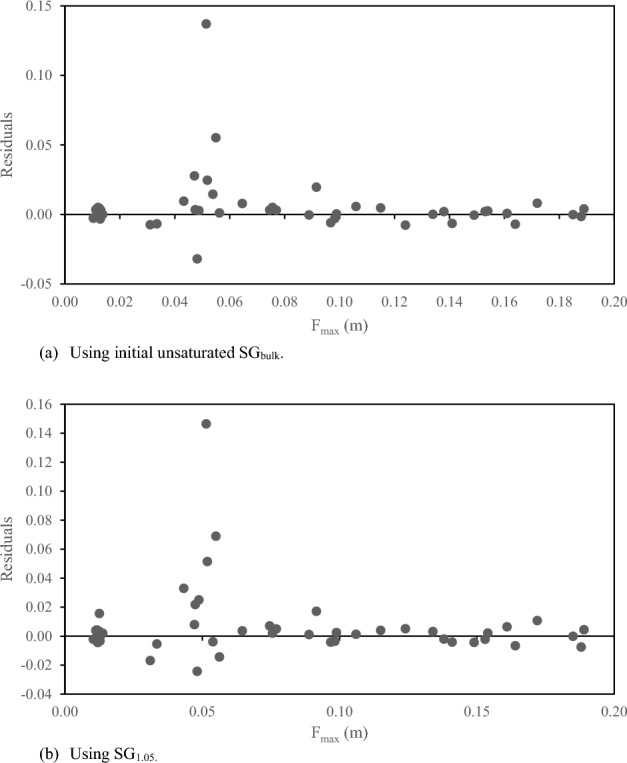
Residuals for predicted vs observed settling velocity using (**a**) initial unsaturated SG_bulk_ and using (**b**) SG_1.05_ plotted against the Feret diameter of each particle.

The residuals of the predicted and the observed settling velocities using the initial unsaturated SG_bulk_ and using SG_1.05_ are shown plotted against F_max_ in Fig. 3. The residuals indicate that the regression is unbiased and homoscedastic, except for residuals with an F_max_ around 0.05 m. Clearly, the regression did not predict settling velocity well for these outlier particles. All the outliers were Norway Maple seeds, which had key differences from the other particles in shape and mass distribution. The Norway Maple seeds were the most elongated particle tested with the highest aspect ratio. Additionally, the Norway Maple seeds had a dense seed at one end of the particle with lighter seed material leading to the other end (see Fig. 2b). All other particles had lower aspect ratios and the American Elm seeds were flat disks with a small seed in the center of disk and therefore had a more even mass distribution.

**Figure 4:**
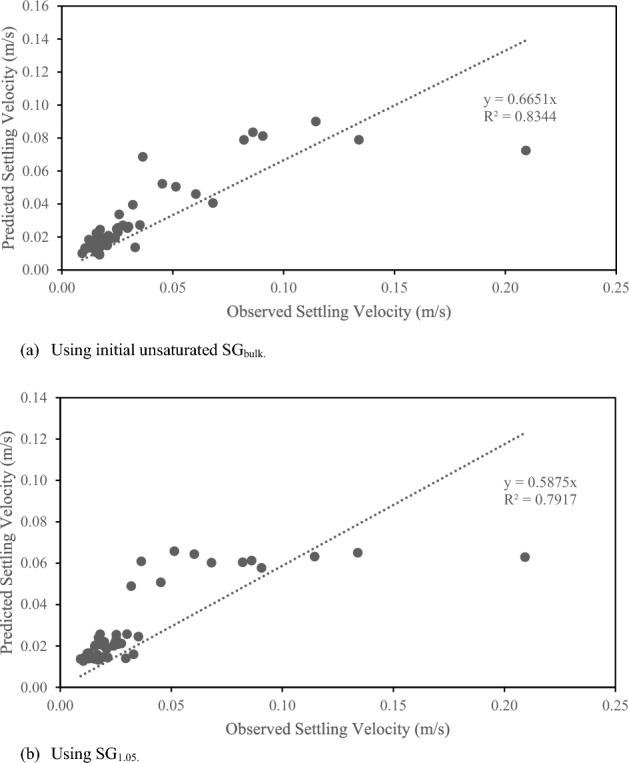
Predicted vs observed settling velocity using (**a**) initial unsaturated SG_bulk_ and using (**b**) SG_1.05_. For both plot (**a**) and plot (**b**), the average C_2_ values by species was used.

The predicted and observed settling velocity values for all 48 particles had a reasonably strong correlation with an R^2^ value of 0.83 and p < 0.05 (Fig. 4a) when using initial SG_bulk_ and R^2^ value of 0.79 when using SG_1.05_ (Fig. 4b). Use of the average C_2_ values do not result in a one-to-one response of the observed and predicted values with the Church equation, suggesting this may not be an appropriate model for these conditions. This methodology does appear to provide reasonable estimates of velocities under 0.08 m/s with less reliability for Norway Maple seeds.

There was no one-to-one relationship found between any of the five measured physical parameters (mass, F_max_, base surface area, SG_bulk_, and displaced water volume) and the average settling velocity for the leaves and seeds of all species. Tables of calculated linear correlation coefficients are included in the Supplementary Information attachment.

### Leaves

For leaves, there was no correlation between any physical parameter and C_2_. A one-way Analysis of Variance (ANOVA) test (α = 0.05) was performed on the two species of leaves (using initial unsaturated SG_bulk_) with the results shown in Table 6. With p = 0.58, the null hypothesis that the two sample averages are equal could not be rejected. With these results, the two species of leaves were grouped together and analyzed (Table 7).

**Table 6:** ANOVA table of C_2_ values for all leaves.

Source of variation	df	SS	MS	F	p	F crit
Between groups	1	2.48 × 10^5^	2.48 × 10^5^	0.317	0.579	4.30
Within groups	22	1.72 × 10^7^	7.81 × 10^5^			
Total	23	1.74 × 10^7^

**Table 7:** C_2_ value statistics for all leaves.

Average	1636
Median	1445
Standard deviation	852
Maximum	3466
Minimum	258

Additional data from more tree species would confirm or falsify that tree leaves have a set range of C_2_ values that are not related to the physical parameters of the individual leaves. For commonly planted boulevard trees, Quercus and Acer species, additional data sets would need to verify results to use the mean C_2_ value to estimate leaf settling velocity, especially with the uncertainty of C_2_ (Table 7).

### Seeds

**Figure 5:**
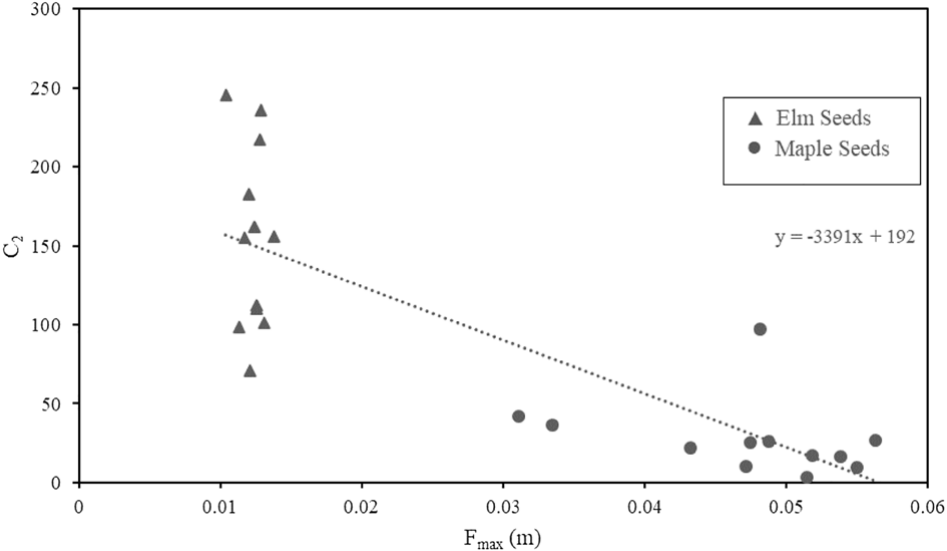
C_2_ vs F_max_ for all seeds tested.

A one-way ANOVA test (α = 0.05) was performed on the two categories of seeds with the results shown in Table 8. Initial unsaturated SG_bulk_ was used in the analysis. With p < 0.05, the null hypothesis that the two averages are equal was rejected. The C_2_ values and settling velocities was consistent within species, but very different in comparison between the two species, as shown statistically in Table 9. There was a correlation between F_max_ and the C_2_ value with R^2^ = 0.67 and p < 0.05 (Fig. 5).

**Table 8:** ANOVA table of C_2_ values for all seeds.

Source of variation	df	SS	MS	F	P-value	F crit
Between groups	1	9.62 × 10^4^	9.62 × 10^4^	49	4.77 × 10^–7^	4.30
Within groups	22	4.29 × 10^4^	1.95 × 10^3^			
Total	23	1.39 × 10^5^

**Table 9:** C_2_ value statistics for seeds.

Statistic	American Elm seeds	Norway Maple seeds
Average	154	27
Median	156	24
Standard deviation	55	23
Maximum	246	97
Minimum	71	3

A lower C_2_ value means the particle falls faster.

The seeds tested were essentially rough spheres with plant tissue surrounding it. It’s likely that the spherical seed controlled the settling velocity and caused the particle to behave more like a natural grain, with Norway Maple seeds having larger seeds, faster settling velocity, and lower C_2_ values than American Elm seeds.

Data from a variety of tree species would be needed to see if the observed relationship between C_2_ and F_max_ holds. If the relationship holds across more species, it could be used to predict a settling velocity of any tree seed based on its F_max_. Otherwise, a set range of C_2_ values could be recorded for each species.

### Bulk specific gravity

**Figure 6:**
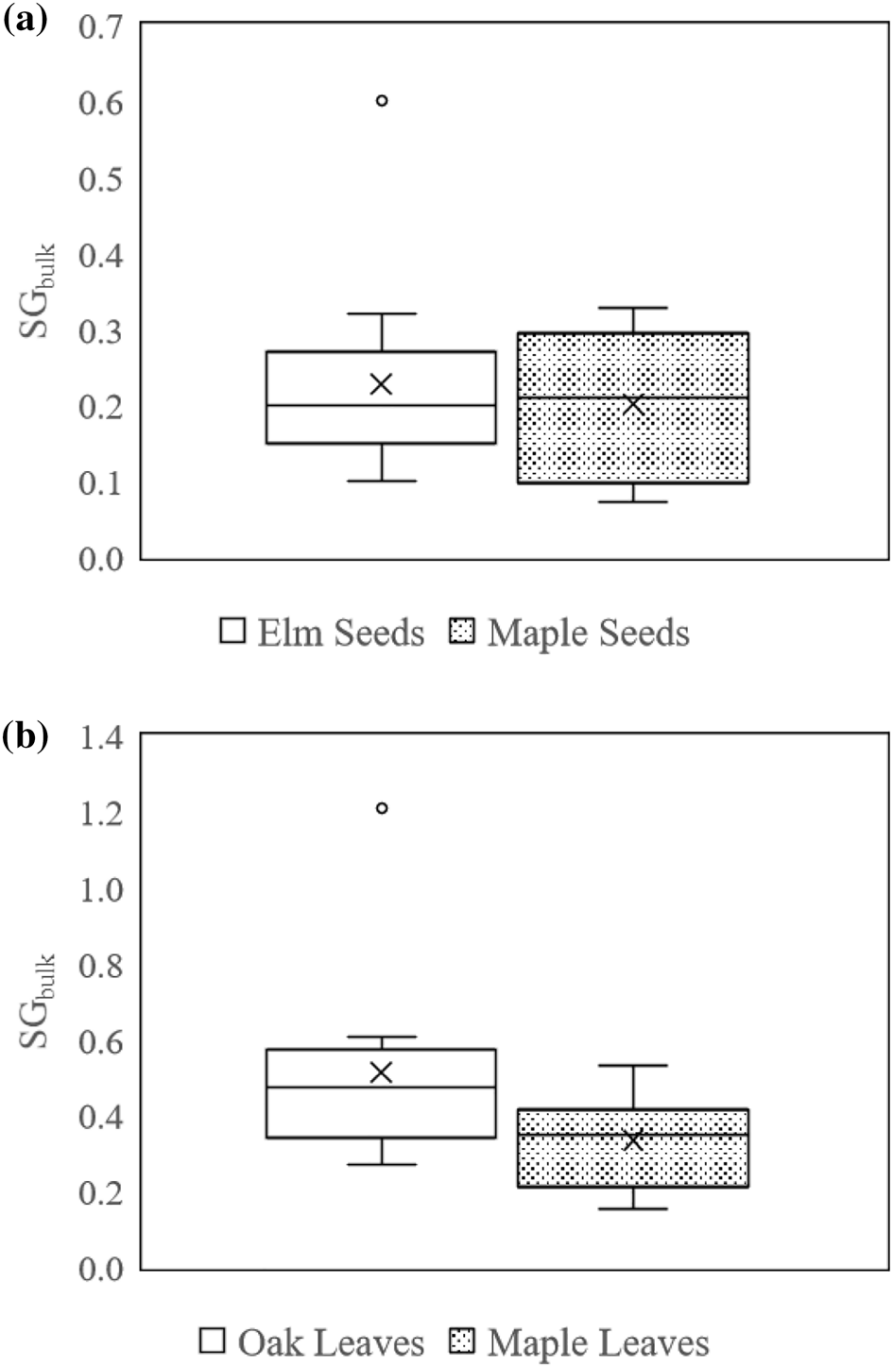
Initial bulk specific gravity box plots for (**a**) tree seeds and (**b**) tree leaves.

Initial unsaturated SG_bulk_ values are presented here and represent the physical properties of the organic particle as they are found in urban settings: on pavement and unsaturated. As previously discussed, particles were saturated prior to the experiments which would result in a saturated SG_bulk_ value closer to 1.05. The measured initial SG_bulk_ for all particle types is summarized in box plots in Fig. 6 and average initial SG_bulk_ values are shown in Table 10. The averages of the initial SG_bulk_ of the two species of leaves were statistically different at α = 0.05 (p = 0.039), but the averages of the two species of seeds were not statistically different at α = 0.05 (p = 0.59). Red Oak leaves had the highest average initial SG_bulk_ of the measured particles, including one initial SG_bulk_ value over one. Karlik and McKay^23^ found similar dry mass density values in Blue Oak leaves, including one leaf with an SG_bulk_ over one. It’s likely that the biological structure and function of the two different tree species caused the difference in initial SG_bulk_, with Red Oak leaves that are generally thicker and retain more water than Norway Maple leaves. Calculation of a fully saturated SG value and partially saturated SG values to reach a SG of 1.05 were consistent in that measurements have each particle able to float prior to saturation and able to sink after saturation as an effective evolutionary dispersal mechanism.

**Table 10:** Initial SG_bulk_ values for all particle types and species.

	American Elm seeds	Norway Maple seeds	Red Oak leaves	Norway Maple leaves
Average	0.23	0.20	0.51	0.33
Median	0.20	0.21	0.48	0.35
Standard deviation	0.13	0.09	0.24	0.13

The two species of tree seeds were not statistically different in terms of measured initial SG_bulk_, but Red Oak leaves had significantly higher inital SG_bulk_ values than Norway Maple leaves.

A one-way ANOVA showed that the sample averages of the initial SG_bulk_ of American Elm seeds and the Norway Maple seeds were not statistically different at α = 0.05 (p = 0.59). A one-way ANOVA showed that the sample averages of initial SG_bulk_ for Red Oak leaves and Norway Maple leaves were statistically different at α = 0.05 (p = 0.039). Comparing all 24 leaf particles to all 24 seed particles, a one-way ANOVA at α = 0.05 (p = 0.0001) indicated that the average initial SG_bulk_ for leaves was significantly different than the average initial SG_bulk_ for seeds. All ANOVA tables relating to initial SG_bulk_ analysis are shown in Table 11.

**Table 11:** ANOVA tests for initial SG_bulk_ values.

Source of variation	df	SS	MS	F	P-value	F crit
American Elm seeds and Norway Maple seeds						
Between groups	1	4.09 × 10^–3^	4.09 × 10^–3^	0.30	0.59	4.30
Within groups	22	0.30	1.35 × 10^–2^			
Total	23	0.30				
Red Oak leaves and Norway Maple leaves						
Between groups	1	0.19	0.19	4.83	3.88 × 10^–2^	4.30
Within groups	22	0.86	3.90 × 10^–2^			
Total	23	1.05				
All seeds and all leaves						
Between groups	1	0.52	0.52	17.9	1.09 × 10^–4^	4.05
Within groups	46	1.35	2.93 × 10^–2^			
Total	47	1.87				

Initial SG_bulk_ values for both species of seeds were generally lower than initial SG_bulk_ values for both species of leaves, and initial SG_bulk_ values between the two species of seeds were very similar. Again, the biological structure of the seeds is likely the cause of the differences. Both species of tree seed are “gliders” that are carried in the wind by delicate webbing surrounding the seed after falling from the tree.

## Conclusions

Settling velocity data for organic nonspherical particles were presented in this study. By solving for the C_2_ drag coefficient in the Ferguson and Church Equation, novel ranges of values for tree leaves and a relationship with F_max_ for tree seeds were given. These results are valuable because they expand upon the wealth of data and analysis available for inorganic settling velocities by exploring the relatively unstudied settling velocities of organic particles.

With additional research, a range of C_2_ values could be assigned to all tree leaves or to several groups of similar tree species based on the resulting data. The C_2_ values presented for American Elm seeds, Red Oak leaves and seeds, and Norway Maple leaves appear to give reasonable settling velocity estimates when using an unsaturated SG value. This methodology does not appear appropriate for the Norway Maple seed, likely due to the asymmetric shape. Machine learning could produce reliable models that predict settling velocity based on plant material shapes. Nutrient content data tied to settling velocity data of organic particles would allow better estimates of stormwater pollutant loading.

This study sets the groundwork for future research on organic particle settling velocity. There are several interesting avenues of research in this area, including, but not limited to: expanding the number of tree species, varying the samples by level of decomposition, and settling the particles in flowing water to simulate stormwater flowing along a curb line. Research on the time it takes for an organic particle to sink in water would be valuable because this information would inform how long the particle floats in stormwater before sinking at the presented settling velocity.

Organic material is known as a driver of nutrient pollution in urban stormwater, but the fate and transport of specific organic particles in the stormwater isn’t well understood. This study is one of the first steps in representing nonspherical organic particles mathematically, which could lead to many practical engineering studies. With a better understanding of the physical properties of stormwater pollutants, cities and governments are better equipped to rehabilitate and sustain their valued water resources.

## Supplementary Information

Supplementary Information.

## Data Availability

Datasets used in this study are included in the Supplementary Information attachment.
