# Supplementary material for: Settling velocities of coarse organic solids
Source: Sci Rep. 2023 Aug 1;13:12436. doi: 10.1038/s41598-023-39645-x (PMC10393945; doi:10.1038/s41598-023-39645-x)
Supplement: Supplementary file 1 — Supplementary Information. [file 41598_2023_39645_MOESM1_ESM.docx]

**Settling Rates of Coarse Organic Solids – Additional Data Attachment**

Aaron J. Pietsch* (piets017@umn.edu), John A. Chapman (chapm155@umn.edu)

University of Minnesota, Twin Cities. Department of Bioproducts and Biosystems Engineering.

1390 Eckles Ave, Saint Paul, MN 55108. United States.

April 2023

1. Physical Properties of Particles

Note: the reported SG values are initial unsaturated SG_bulk_ values.

| Particle Type | ID | Mass (g) | F_max_ (m) | Base Surface Area (m^2^) | SG | Displaced Water Vol (m^3^) |
| --- | --- | --- | --- | --- | --- | --- |
| Maple Seed | MS1 | 0.157 | 0.0550 | 5.76E-04 | 0.24 | 6.60E-07 |
|  | MS2 | 0.033 | 0.0539 | 5.49E-04 | 0.08 | 4.00E-07 |
|  | MS3 | 0.038 | 0.0563 | 5.68E-04 | 0.10 | 4.00E-07 |
|  | MS4 | 0.022 | 0.0472 | 4.01E-04 | 0.07 | 3.00E-07 |
|  | MS5 | 0.012 | 0.0335 | 2.23E-04 | 0.17 | 7.00E-08 |
|  | MS6 | 0.107 | 0.0515 | 4.64E-04 | 0.21 | 5.00E-07 |
|  | MS7 | 0.090 | 0.0488 | 3.63E-04 | 0.30 | 3.00E-07 |
|  | MS8 | 0.110 | 0.0475 | 4.41E-04 | 0.28 | 4.00E-07 |
|  | MS9 | 0.128 | 0.0519 | 5.17E-04 | 0.33 | 3.90E-07 |
|  | MS10 | 0.082 | 0.0482 | 4.62E-04 | 0.21 | 4.00E-07 |
|  | MS11 | 0.018 | 0.0311 | 2.24E-04 | 0.11 | 1.70E-07 |
|  | MS12 | 0.064 | 0.0433 | 3.33E-04 | 0.32 | 2.00E-07 |
| Elm Seed | ES1 | 0.008 | 0.0117 | 5.78E-05 | 0.32 | 2.50E-08 |
|  | ES2 | 0.005 | 0.0113 | 4.85E-05 | 0.20 | 2.50E-08 |
|  | ES3 | 0.006 | 0.0138 | 6.98E-05 | 0.24 | 2.50E-08 |
|  | ES4 | 0.006 | 0.0126 | 4.45E-05 | 0.20 | 3.00E-08 |
|  | ES5 | 0.006 | 0.0126 | 5.82E-05 | 0.60 | 1.00E-08 |
|  | ES6 | 0.002 | 0.0104 | 3.88E-05 | 0.20 | 1.00E-08 |
|  | ES7 | 0.015 | 0.0128 | 7.09E-05 | 0.15 | 1.00E-07 |
|  | ES8 | 0.014 | 0.0129 | 6.88E-05 | 0.28 | 5.00E-08 |
|  | ES9 | 0.015 | 0.0131 | 6.43E-05 | 0.15 | 1.00E-07 |
|  | ES10 | 0.011 | 0.0121 | 5.80E-05 | 0.11 | 1.00E-07 |
|  | ES11 | 0.008 | 0.0120 | 5.66E-05 | 0.10 | 8.00E-08 |
|  | ES12 | 0.012 | 0.0124 | 5.51E-05 | 0.17 | 7.00E-08 |

| Particle Type | ID | Mass (g) | F_max_ (m) | Base Surface Area (m^2^) | SG | Displaced Water Vol (m^3^) |
| --- | --- | --- | --- | --- | --- | --- |
| Red Oak Leaf | OL1 | 0.597 | 0.188 | 6.68E-03 | 0.27 | 2.20E-06 |
|  | OL2 | 0.527 | 0.138 | 4.03E-03 | 0.31 | 1.70E-06 |
|  | OL3 | 0.246 | 0.164 | 3.77E-03 | 0.48 | 5.10E-07 |
|  | OL4 | 0.213 | 0.115 | 2.63E-03 | 0.43 | 4.90E-07 |
|  | OL5 | 0.115 | 0.0989 | 2.29E-03 | 0.58 | 2.00E-07 |
|  | OL6 | 0.304 | 0.134 | 2.89E-03 | 0.61 | 5.00E-07 |
|  | OL7 | 0.27 | 0.154 | 4.00E-03 | 0.45 | 6.00E-07 |
|  | OL8 | 0.458 | 0.172 | 5.47E-03 | 0.57 | 8.00E-07 |
|  | OL9 | 0.514 | 0.185 | 8.08E-03 | 0.47 | 1.10E-06 |
|  | OL10 | 0.725 | 0.189 | 8.31E-03 | 0.48 | 1.50E-06 |
|  | OL11 | 0.165 | 0.106 | 2.13E-03 | 0.28 | 6.00E-07 |
|  | OL12 | 0.266 | 0.124 | 3.24E-03 | 1.21 | 2.20E-07 |
| Norway Maple Leaf | ML1 | 0.124 | 0.0667 | 1.67E-03 | 0.21 | 6.00E-07 |
|  | ML2 | 0.415 | 0.153 | 6.37E-03 | 0.21 | 2.00E-06 |
|  | ML3 | 0.215 | 0.149 | 3.22E-03 | 0.22 | 1.00E-06 |
|  | ML4 | 0.189 | 0.0916 | 3.14E-03 | 0.24 | 8.00E-07 |
|  | ML5 | 0.046 | 0.0646 | 8.42E-04 | 0.15 | 3.00E-07 |
|  | ML6 | 0.125 | 0.0770 | 1.66E-03 | 0.42 | 3.00E-07 |
|  | ML7 | 0.073 | 0.0968 | 1.41E-03 | 0.41 | 1.80E-07 |
|  | ML8 | 0.053 | 0.0746 | 1.10E-03 | 0.53 | 1.00E-07 |
|  | ML9 | 0.328 | 0.141 | 5.14E-03 | 0.41 | 8.00E-07 |
|  | ML10 | 0.319 | 0.161 | 6.27E-03 | 0.53 | 6.00E-07 |
|  | ML11 | 0.184 | 0.0983 | 4.33E-03 | 0.31 | 6.00E-07 |
|  | ML12 | 0.196 | 0.0889 | 2.10E-03 | 0.39 | 5.00E-07 |

| Particle Type | ID | Mass (g) | F_max_ (m) | Base Surface Area (m^2^) | SG | Displaced Water Vol (m^3^) |
| --- | --- | --- | --- | --- | --- | --- |
| Wood Chip | WC1 | 0.04 | 0.0237 | 8.55E-05 | 0.44 | 9.00E-08 |
|  | WC2 | 0.16 | 0.0228 | 1.38E-04 | 0.53 | 3.00E-07 |
|  | WC3 | 0.03 | 0.0124 | 3.66E-05 | 0.60 | 5.00E-08 |
|  | WC4 | 0.12 | 0.0371 | 1.52E-04 | 0.60 | 2.00E-07 |
|  | WC5 | 0.04 | 0.0175 | 4.78E-05 | 0.40 | 1.00E-07 |
|  | WC6 | 0.18 | 0.0343 | 1.47E-04 | 0.51 | 3.50E-07 |
|  | WC7 | 0.07 | 0.0124 | 5.30E-05 | 0.28 | 2.50E-07 |
|  | WC8 | 0.11 | 0.0294 | 1.06E-04 | 0.37 | 3.00E-07 |

1. Settling Velocity Trials and Averages

| Particle Type | ID | Settling Velocity – Trial 1 (m/s) | Settling Velocity – Trial 2 (m/s) | Settling Velocity– Trial 3 (m/s) | Average Settling Velocity (m/s) |
| --- | --- | --- | --- | --- | --- |
| Maple Seed | MS1 | 0.1370 | 0.1510 | 0.1140 | 0.1340 |
|  | MS2 | 0.0523 | 0.0398 | 0.0892 | 0.0604 |
|  | MS3 | 0.0423 | 0.0543 | 0.0577 | 0.0514 |
|  | MS4 | 0.0820 | 0.0573 | 0.0652 | 0.0682 |
|  | MS5 | 0.0657 | 0.0399 | 0.0304 | 0.0453 |
|  | MS6 | 0.2860 | 0.2170 | 0.1250 | 0.2093 |
|  | MS7 | 0.0740 | 0.0956 | 0.0890 | 0.0862 |
|  | MS8 | 0.0530 | 0.1150 | 0.0785 | 0.0822 |
|  | MS9 | 0.0480 | 0.0907 | 0.2050 | 0.1146 |
|  | MS10 | 0.0361 | 0.0385 | 0.0350 | 0.0365 |
|  | MS11 | 0.0340 | 0.0350 | 0.0270 | 0.0320 |
|  | MS12 | 0.0530 | 0.0810 | 0.1380 | 0.0907 |
| Elm Seed | ES1 | 0.0237 | 0.0171 | 0.0122 | 0.0177 |
|  | ES2 | 0.0187 | 0.0120 | 0.0208 | 0.0172 |
|  | ES3 | 0.0234 | 0.0146 | 0.0118 | 0.0166 |
|  | ES4 | 0.0167 | 0.0186 | 0.0163 | 0.0172 |
|  | ES5 | 0.0285 | 0.0199 | 0.0403 | 0.0296 |
|  | ES6 | 0.0099 | 0.0107 | 0.0108 | 0.0105 |
|  | ES7 | 0.0117 | 0.0097 | 0.0107 | 0.0107 |
|  | ES8 | 0.0114 | 0.0179 | 0.0130 | 0.0141 |
|  | ES9 | 0.0259 | 0.0133 | 0.0082 | 0.0158 |
|  | ES10 | 0.0189 | 0.0137 | 0.0139 | 0.0155 |
|  | ES11 | 0.0077 | 0.0080 | 0.0119 | 0.0092 |
|  | ES12 | 0.0132 | 0.0107 | 0.0152 | 0.0130 |

| Particle Type | ID | Settling Velocity – Trial 1 (m/s) | Settling Velocity – Trial 2 (m/s) | Settling Velocity – Trial 3 (m/s) | Average Settling Velocity (m/s) |
| --- | --- | --- | --- | --- | --- |
| Red Oak Leaf | OL1 | 0.0253 | 0.0139 | 0.0149 | 0.0180 |
|  | OL2 | 0.0205 | 0.0166 | 0.0226 | 0.0199 |
|  | OL3 | 0.0074 | 0.0210 | 0.0234 | 0.0173 |
|  | OL4 | 0.0226 | 0.0242 | 0.0253 | 0.0240 |
|  | OL5 | 0.0207 | 0.0291 | 0.0132 | 0.0210 |
|  | OL6 | 0.0225 | 0.0098 | 0.0420 | 0.0248 |
|  | OL7 | 0.0378 | 0.0190 | 0.0190 | 0.0253 |
|  | OL8 | 0.0358 | 0.0270 | 0.0429 | 0.0352 |
|  | OL9 | 0.0300 | 0.0346 | 0.0113 | 0.0253 |
|  | OL10 | 0.0268 | 0.0211 | 0.0424 | 0.0301 |
|  | OL11 | 0.0231 | 0.0239 | 0.0144 | 0.0205 |
|  | OL12 | 0.0382 | 0.0256 | 0.0138 | 0.0259 |
| Norway Maple Leaf | ML1 | 0.0118 | 0.0208 | 0.017 | 0.0165 |
|  | ML2 | 0.0108 | 0.0223 | 0.0221 | 0.0184 |
|  | ML3 | 0.0128 | 0.0244 | 0.0106 | 0.0159 |
|  | ML4 | 0.0321 | 0.0356 | 0.0315 | 0.0331 |
|  | ML5 | 0.0191 | 0.0181 | 0.0138 | 0.0170 |
|  | ML6 | 0.0224 | 0.014 | 0.0223 | 0.0196 |
|  | ML7 | 0.0081 | 0.021 | 0.0076 | 0.0122 |
|  | ML8 | 0.0185 | 0.0138 | 0.032 | 0.0214 |
|  | ML9 | 0.0106 | 0.0197 | 0.0165 | 0.0156 |
|  | ML10 | 0.0317 | 0.0252 | 0.0258 | 0.0276 |
|  | ML11 | 0.0136 | 0.0143 | 0.0111 | 0.0130 |
|  | ML12 | 0.0162 | 0.0239 | 0.0102 | 0.0168 |

| Particle Type | ID | Settling Velocity – Trial 1 (m/s) | Settling Velocity – Trial 2 (m/s) | Settling Velocity – Trial 3 (m/s) | Average Settling Velocity (m/s) |
| --- | --- | --- | --- | --- | --- |
| Wood Chip | WC1 | 0.0076 | 0.0177 | 0.0159 | 0.0137 |
|  | WC2 | 0.0813 | 0.0691 | 0.0619 | 0.0708 |
|  | WC3 | 0.0580 | 0.0998 | 0.0536 | 0.0705 |
|  | WC4 | 0.1400 | 0.0718 | 0.0921 | 0.1013 |
|  | WC5 | 0.0651 | 0.0719 | 0.1100 | 0.0823 |
|  | WC6 | 0.1260 | 0.1130 | 0.1170 | 0.1187 |
|  | WC7 | 0.1280 | 0.1250 | 0.1480 | 0.1337 |
|  | WC8 | 0.0704 | 0.0844 | 0.0842 | 0.0797 |

1. Correlation Tables

|  | Average Settling Velocity (m/s) | Mass (g) | SG | Displaced Water Vol (m^3^) | F_max_ (m) | Base SA (m^2^) |
| --- | --- | --- | --- | --- | --- | --- |
| Average Settling Velocity (m/s) | 1.00 |  |  |  |  |  |
| Mass (g) | 0.83 | 1.00 |  |  |  |  |
| SG | 0.14 | 0.20 | 1.00 |  |  |  |
| Displaced Water Vol (m^3^) | 0.77 | 0.78 | -0.09 | 1.00 |  |  |
| F_max_ (m) | 0.75 | 0.79 | -0.10 | 0.99 | 1.00 |  |
| Base SA (m^2^) | 0.80 | 0.88 | -0.12 | 0.91 | 0.94 | 1.00 |

|  | Average Settling Velocity (m/s) | Mass (g) | SG | Displaced Water Vol (m^3^) | F_max_ (m) | Base SA (m^2^) |
| --- | --- | --- | --- | --- | --- | --- |
| Average Settling Velocity (m/s) | 1.00 |  |  |  |  |  |
| Mass (g) | 0.40 | 1.00 |  |  |  |  |
| SG | 0.39 | 0.08 | 1.00 |  |  |  |
| Displaced Water Vol (m^3^) | 0.36 | 0.86 | 0.17 | 1.00 |  |  |
| F_max_ (m) | 0.37 | 0.89 | 0.06 | 0.88 | 1.00 |  |
| Base SA (m^2^) | 0.06 | 0.81 | -0.37 | 0.64 | 0.71 | 1.00 |
